# Correlates of quality of life of pre-obese and obese patients: a pharmacy-based cross-sectional survey

**DOI:** 10.1186/1471-2458-9-337

**Published:** 2009-09-15

**Authors:** Laurent Laforest, Eric Van Ganse, Cécile Ritleng, Gaelle Desamericq, Laurent Letrilliart, Alain Moreau, Sarah Rosen, Hubert Mechin, Genevieve Chamba

**Affiliations:** 1 Unité de Neuroépidémiologie et de Pharmacoépidémiologie, Service Neurologie, CHU Lyon, Bron, France; 2 Département de médecine générale, Université Claude Bernard Lyon 1, Lyon, France; 3 MAPI group, MAPI SAS, Lyon, France; 4 Pharmakeion, Lyon, France

## Abstract

**Background:**

The correlates of quality of life (QOL), as measured by the OSQOL questionnaire were investigated in a convenience sample of overweight patients recruited in pharmacies.

**Methods:**

A convenience sample of patients with a Body Mass Index ≥ 28 kg/m^2 ^were recruited in community-based pharmacies. Baseline characteristics and QOL dimensions (1-Physical state, 2-Vitality-desire to do things, 3-Relations with others, 4-Psychological state) were reported in self-completed questionnaires from which the risk of obtaining a low QOL was assessed for each dimension.

**Results:**

QOL was inadequate for all dimensions in the 494 patients included in the study (median age = 61, 48% women, 21% professional persons/top executives). Older pre-obese and obese patients were more likely to report impaired physical functioning (OR = 2.02, 95%CI = [1.10-3.70]), but were less severely affected socially (OR = 0.32, 95%CI = [0.15-0.69]). Pre-obese and obese professional persons and top executives showed better physical capabilities (OR = 0.35, 95%CI = [0.15-0.81]) and increased vitality (OR = 0.47, 95%CI = [0.23-0.95]). Overall, men's psychological state was better than females' (OR = 0.46, 95%CI = [0.25-0.82]). A body-mass index ≥ 35 kg/m^2 ^was significantly associated with poorer QOL scores on physical, relational and psychological dimensions.

**Conclusion:**

Our data highlighted the influence of the severity of excess weight, gender, age and socioeconomic status on QOL. These factors should be taken into account when interpreting QOL in pre-obese and obese persons.

## Background

Studies have suggested an increased prevalence of obesity throughout the world over the past years [1], particularly in Western countries [2-4] including France [5,6]. The consequences of obesity and more generally excess weight on mortality and morbidity [7,8] notably cardiovascular diseases are well established [9]. Likewise, the consequences on osteo-articular and endocrinal morbidity should not be overlooked [10].

Quality of life (QOL) gave rise to an ongoing interest these past years. QOL is a major tool to estimate patients' perceived burden of diseases, for research purposes as well as for medical practice [11]. It has become a common end-point in clinical trials, along with clinical outcomes. In the absence of perspective of recovery, QOL remains a useful criterion in the management of chronic diseases [11].

Obesity may also have detrimental consequences on patients' health-related QOL, particularly their physical functioning [12-14]. In contrast, the impact of obesity on mental components of QOL yielded more controversial conclusions [12-15].

Increasing our existing awareness of factors influencing QOL in this population may be helpful in terms of public health. Indeed the potential identification of sub-groups of patients with poor QOL may be a preliminary step before implementing preventive action for improved management of overweight and obesity. Hence, the impact of personal characteristics, body mass index (BMI), disease-related factors and health habits of pre-obese and obese persons on different QOL dimensions should be better explored. In addition, it is unclear to which extent the relationships between BMI and QOL scores are influenced by other factors.

The aim of the present pharmacy-based study was thus to identify the correlates of a poor quality of life, based on different dimensions, in a population of overweight persons, using a specific QOL questionnaire (OSQOL). We also investigated whether the relationship between OSQOL scores and BMI varied according to patients' other characteristics.

## Methods

### Study design and population

A survey was conducted in 2005 in 76 French community-based pharmacies of the Rhone-Alpes Region. A convenience sample of patients with probable excess weight visiting the study pharmacies was consecutively recruited. Patients were asked to participate in the study. A prerequisite was to be a regular customer of the pharmacy (at least 12 months of dispensed drugs recorded in the computerized database of the pharmacy). Once the objectives of the study had been explained, patients accepting to participate were asked to complete a self-completed questionnaire. Whenever possible, pharmacies were asked to recruit an equal number of patients over and under 60, to ensure sufficient age variation. The study was approved by the French National Regulatory Body (Commission Nationale Informatique et Libertés -CNIL).

### Data collected

Data were obtained from self-completed questionnaires. Self-completed questionnaires consisted of data on baseline characteristics, including socio-professional status: top executives/professional persons (upper-social class), workmen/employees, teachers/artisans/shopkeepers and unemployed/housewives-husbands. Retired patients were asked to report their last professional position. Patients were also asked to state their previous efforts to change their lifestyle (none, minor, substantial, major change) with physical exercise and diet, with the aim of improving their health. Patients reported their height and weight in questionnaires so that their BMI could be computed when data were analysed.

QOL was measured using the Obesity Specific Quality Of Life (OSQOL) questionnaire [16]. This disease-specific questionnaire includes 11 questions grouped into four dimensions: 1- "Physical state" (7 questions), 2- "Vitality, desire to do things" (2 questions), 3- "Relations with other people" (1 question), 4- "Psychological state" (1 question). Each question offered five possible answers ('absolutely false', 'fairly false', 'neither true nor false', 'fairly true' and 'absolutely true'), classified according to reduced QOL. For dimensions 1 and 2, a quantitative score was calculated (0% minimal QOL, 100% maximal QOL) [16].

Co-morbid diagnoses were identified from drug therapies dispensed over the past 12 months and classified according to the Anatomical Therapeutic and Chemical Classification. Co-morbid diagnoses included angina pectoris (C01DA, C07 and C08), diabetes mellitus (A10), hypertension (C02, C03, C07, C08 and C09), heart failure (C01AA, C01B, C03, C07 and C09), dyslipidemia (C10), rheumatic conditions (M01), dysthyroidism (H03), gastrointestinal disease (A02) and asthma/COPD (R03). In case of isolated unspecific therapy, pharmacists were oriented by patient's medical history.

### Analyses

Analyses were conducted only on patients with BMI ≥ 28 kg/m^2 ^and who had completed all dimensions of their OSQOL questionnaire.

The objective was to compare patients with a poor QOL to the rest of the survey population for each QOL dimension, according to personal and medical characteristics and reported efforts to change lifestyle habits.

Due to skewed distributions, quantitative scores for dimensions 1 and 2 were dichotomized according to their respective lowest quartile values (no transformation permitted to normalize distributions). A score not greater than the 25%-quartile value indicated a poor QOL. For dimensions 3 and 4 (which included a single item), patients were considered to have a poor QOL if they answered 'fairly true' or 'completely true' to the question. The Chi-square test was used for univariate analyses.

In addition, multivariate logistic models were programmed. For each dichotomized QOL dimension, the risk of obtaining results reflecting a poor QOL was assessed. All models were adjusted for age, gender, BMI and socio-economic status. Other covariates were included if a significant univariate association was established with the corresponding studied variable (p < 0.10).  Complementary analyses were also conducted to assess to which extent the statistical relationship between BMI and the different OSQOL scores varied according to other patients' characteristics. Interactions between OSQOL scores, BMI and the different co-factors were also tested. Corresponding results would be reported only when relevant.  Analyses were conducted with SAS software.

## Results

### 1- Patients' characteristics

579 patients with probable overweight and regular customers of the pharmacies accepted to complete the self-questionnaire. Two questionnaires were not returned by the patients. Other patients were excluded before analyses as BMI was lower than 28 kg/m^2 ^(n = 25) or not documented (n = 1). Among the 551 remaining patients, 494 completed all OSQOL dimensions. The 57 patients excluded from the analyses were older (p = 0.02) but showed no difference from the included population with regards to BMI (p = 0.50), gender (p = 0.98) and socio-professional status (p = 0.90).

The median age of the 494 patients was 61 years old (range 27-86, 25^th^-75^th ^percentiles: 54-72), almost half of them were females and the BMI varied from 28 to 51 kg/m^2 ^(median value: 32 kg/m^2^). About one in five patients was a top executive or a professional person (Table 1). The median number of co-morbid diagnoses was three (range 0-7) with the most common co-morbid conditions being hypertension (72.7%), diabetes (36.4%) and dyslipidemia (51.8%). Few patients reported having made substantial or major changes in dietary habits and physical activity with the aim of improving their health status (Table 1).

**Table 1:** Patient characteristics (n = 494)

**Age (years)**	**n**	**%**
< 60	217	43.9
60 - 69.9	123	24.9
≥ 70	154	31.2
Gender		
Males	259	52.4
Females	235	47.6

BMI (kg/m^2^)		
28-29.9	154	31.2
30 - 34.9	186	37.6
≥ 35	154	31.2

Socio-economic status		
Workmen -employees	213	49.1
Top executives -Professional persons	92	21.2
Teachers - artisans shopkeepers	63	14.5
Unemployed-Housewives/husbands	66	15.2

Current smoker		
No	415	87.7
Yes	58	12.3

Alcohol		
None	269	55.3
1-2 glasses per day	134	27.6
≥ 3 glasses per day	83	17.1

Number of co-morbid diagnoses		
None or one	78	15.8
Two	127	25.7
Three or more	289	58.5

Previous efforts for substantial changes in dietary habits		
Yes	180	37.7
No	298	62.3

Previous efforts for substantial changes in physical activities		
Yes	79	17.0
No	386	83.0

Counts that do not add to 494 are due to missing data

### 2- Quality of life scores

Detailed answers to the OSQOL questionnaire are reported in Table 2. Physical limitations perceived by patients or other were commonly reported (Dimension 1, Table 2). The influence of excess weight on psychological and relational dimensions was also significant: a noticeable proportion of patients reported that they felt ill-at-ease due to excess weight or obesity (22.3% "absolutely" or "fairly true"), or attacked when people talked about their weight (19.6% "absolutely" or "fairly true"). Median values of quantitative scores for dimensions 1 and 2 were 52.5% (25^th^-75^th ^percentiles: 39.4%-73.0%) and 57.9% (25^th^-75^th ^percentiles: 41.0%-81.6%), respectively.

**Table 2:** Patients' quality of life (OSQOL questionnaire, n = 494)

	**Absolutely true %**	**Fairly true** **%**	**Neither true, nor false %**	**Fairly false %**	**Absolutely false %**
**Dimension 1: Physical state**					
1- I have trouble squatting	30.2	32.2	9.9	10.7	17.0
2- I cannot sit down in a very low armchair	21.7	26.3	11.3	11.9	28.7
3- I walk as little as possible	9.3	14.2	10.9	25.9	39.7
4- I have to stop to catch my breath after walking several hundred meters	11.7	14.4	9.5	22.3	42.1
5- I have trouble climbing stairs	16.2	30.4	9.3	21.0	23.1
6- People say I am not very athletic	32.6	25.9	18.4	9.7	13.4
7- People often say that I am not agile	12.7	21.9	24.9	20.8	19.6

**Dimension 2: Vitality desire to do things**					
8- I often lack energy	8.9	29.1	14.8	22.9	24.3
9- I do not move around very much	9.7	21.3	12.7	25.9	30.4

**Dimension 3: Relations with others**					
10- I feel I am being attacked when people talk about my weight	7.3	12.3	17.2	21.5	41.7

**Dimension 4: Psychological state**					
11- I feel very ill-at-ease	7.7	14.6	16.4	18.8	42.5

### 3- Univariate correlates

The study showed that patients' QOL scores significantly deteriorated with increasing BMI (notably in case of severe obesity), even though the statistical association was less marked for Vitality scores (Table 3). Age had no significant influence on dimensions 1, 2 and 4. In contrast, pre-obese and obese patients under 60 were more affected in their relations with others. On the whole, women exhibited a worse QOL level than men for physical, relational and psychological dimensions. Significant differences were observed according to socio-economic status for physical state, vitality and psychological state, with top executives and professional persons achieving better results for these dimensions compared to other socio-economic categories. Differences were less marked for dimension 3 (Table 3).

**Table 3:** OSQOL Univariate results

		**Dimension 1** **Physical state**		**Dimension 2:** **Vitality desire to do things**		**Dimension 3** Relations with others * "I feel I am being attacked when people talk about my weight"		**Dimension 4** Psychological state ** "I feel very ill-at-ease "	
**Variables**	**n**	**% patients** **≤ Q25% score (1)**	**p**	**% patients ≤ Q25% score (1)**	**p**	**% 'Fairly true' or 'absolutely true'**	**p**	**% 'Fairly true' or 'absolutely true'**	**p**

Overall	494	25.1		27.9		19.6		22.3	
									
**Age (years)**			0.168		0.837		0.002		0.108
**< 60**	217	21.7		29.0		26.3		26.7	
**60 - 69.9**	123	30.9		26.0		17.9		18.7	
**≥ 70**	154	25.3		27.9		11.7		18.8	
									
**Gender**			0.007		0.202		0.002		<.0001
Males	259	20.1		25.5		14.3		13.9	
Females	235	30.6		30.6		25.5		31.5	
									
**Body Mass Index (kg/m^2^)**			<.0001		0.028		<.0001		<.0001
28-29.9	154	12.3		22.7		13.6		13.0	
30 - 34.9	186	21.5		25.8		15.1		19.3	
≥ 35	154	42.2		35.7		31.2		35.1	
									
**Socio-economic status**			0.002		0.027		0.082		0.001
Workmen - employees	213	27.2		31.9		20.2		25.3	
Top executives -Professional persons	92	9.8		15.2		13.0		8.7	
Teachers - artisans - shopkeeper	63	27.0		28.6		15.9		17.5	
Unemployed/housewives-husbands	66	33.3		28.8		28.8		31.8	
									
**Current smoking**			0.620		0.041		0.037		0.357
No	415	24.6		26.7		17.8		22.2	
Yes	58	27.6		39.7		29.3		27.6	
									
**Alcohol**			0.251		0.791		0.017		0.004
None	269	28.2		26.8		24.2		27.1	
1-2 glasses per day	134	21.6		29.1		14.2		18.7	
≥ 3 glasses per day	83	21.7		30.1		13.2		10.8	
									
**Number of associated co-morbid diagnoses**			0.018		0.020		0.479		0.933
None or one	78	17.9		24.4		16.7		21.8	
Two	127	18.9		19.7		17.3		21.3	
Three or more	289	29.8		32.5		21.4		22.8	
									
**Previous efforts for substantial changes in dietary habits**			0.487		0.117		0.216		0.947
No'	298	26.2		30.5		18.1		22.5	
Yes	180	23.3		23.9		22.8		22.2	
									
**Previous efforts for substantial changes in physical activities**			0.027		0.019		0.381		0.958
No	386	26.9		30.8		19.7		23.1	
Yes	79	15.2		17.7		24.0		22.8	

(1) Q25% = first quartile- A score ≤ Q25% indicates a poor QOL, and a high QOL otherwise. Chi-squared tests were used for all statistical comparisons

Patients who reported previous substantial efforts in exercising to improve health had significantly better QOL results for dimensions 1 and 2. In contrast, reported efforts on diet had a limited impact. An association was observed between alcohol consumption and dimensions 3 and 4 and smoking was found to have a significant effect on dimensions 2 and 3.

Finally, the number of co-morbid diagnoses had a significant impact on physical functioning and vitality dimensions, notably beyond two associated diagnoses (Table 3). When the category "3 or more co-morbid diagnoses" was detailed into "3" and "4 or more", these conclusions were not affected (data not shown).

#### Statistical relationships between OSQOL scores and BMI according to other factors

The relationships between BMI and the different OSQOL dimensions according to the other factors are detailed in Table 4. Interactions tested between OSQOL scores, BMI and the different other co-factors did not reach significance threshold (data not shown), except for vitality score and BMI according to patient's previous efforts to change dietary habits (p = 0.02). However, this interaction is difficult to interpret in practical terms (Table 4).

**Table 4:** Relationship between OSQOL dimensions and BMI according to the other variables

		**Dimension 1**		**Dimension 2**		**Dimension 3**		**Dimension 4**	
		**Physical state**		**Vitality desire to do things**		**Relations with others (1)**		**Psychological state (2)**	
		**%**							
	**n**	**≤ Q25% score (3)**	**p**	**% ≤ Q25% score (3)**	**p**	**% 'Fairly true' or 'absolutely true'**	**p**	**% 'Fairly true' or 'absolutely true'**	**p**

**BMI (kg/m^2^) OVERALL**									
28-29.9	154	12.3		22.7		13.6		13.0	
30 - 34.9	186	21.5		25.8		15.1		19.3	
≥ 35	154	42.2		35.7		31.2		35.1	
									
**Age (years)**									
**< 60**			<.0001		0.2268		0.0481		0.009
28-29.9 kg/m^2^	53	3.8		20.8		20.8		13.2	
30 - 34.9 kg/m^2^	83	19.3		28.9		20.5		25.3	
≥ 35 kg/m^2^	81	35.8		34.6		35.8		37.0	
**60 - 69.9**			<.0001		0.0227		0.0286		0.005
28-29.9 kg/m^2^	38	7.9		21.1		13.2		5.3	
30 - 34.9 kg/m^2^	47	27.7		17.0		10.6		17.0	
≥ 35 kg/m^2^	38	57.9		42.1		31.6		34.2	
**≥ 70**			0.072		0.8085		0.2447		0.075
28-29.9 kg/m^2^	63	22.2		25.4		7.9		17.5	
30 - 34.9 kg/m^2^	56	19.6		28.6		10.7		12.5	
≥ 35 kg/m^2^	35	40.0		31.4		20.0		31.4	
									
**Gender**									
**Males**			<.0001		0.298		0.0993		0.0176
28-29.9 kg/m^2^	84	8.3		22.6		9.5		7.1	
30 - 34.9 kg/m^2^	111	15.3		23.4		13.5		13.5	
≥ 35 kg/m^2^	64	43.8		32.8		21.9		23.4	
**Females**			0.0049		0.1217		0.0031		0.0051
28-29.9 kg/m^2^	70	17.1		22.9		18.6		20.0	
30 - 34.9 kg/m^2^	75	30.7		29.3		17.3		28.0	
≥ 35 kg/m^2^	90	41.1		37.8		37.8		43.3	
									
**Socio-economic status**									
**Top executives -Professional persons**			<.0001		0.2642		0.0207		0.0873
28-29.9 kg/m^2^	36	0.0		11.1		5.6		5.6	
30 - 34.9 kg/m^2^	38	5.3		13.2		10.5		5.3	
≥ 35 kg/m^2^	18	38.9		27.8		33.3		22.2	
**Other classes**			0.0005		0.4219		0.0029		0.0014
28-29.9 kg/m^2^	98	17.4		26.5		14.3		14.3	
30 - 34.9 kg/m^2^	126	25.4		30.2		16.7		23.8	
≥ 35 kg/m^2^	118	40.7		34.8		31.4		35.6	
**Current smoking**									
**No**			<.0001		0.1034		<.0001		0.0006
28-29.9 kg/m^2^	134	13.4		22.4		9.7		14.2	
30 - 34.9 kg/m^2^	156	21.2		25.0		15.4		19.9	
≥ 35 kg/m^2^	125	40.8		33.6		29.6		33.6	
**Yes**			0.0005		0.1067		0.0613		0.0115
28-29.9 kg/m^2^	16	0.0		18.8		31.3		6.3	
30 - 34.9 kg/m^2^	19	21.1		42.1		10.5		21.1	
≥ 35 kg/m^2^	23	52.2		52.2		43.5		47.8	
									
**Alcohol**									
**No**			0.0002		0.2312		0.0057		0.0006
28-29.9 kg/m^2^	77	15.6		20.8		18.2		15.6	
30 - 34.9 kg/m^2^	96	24.0		26.0		17.7		22.9	
≥ 35 kg/m^2^	96	42.7		32.3		35.4		40.6	
**Yes**			<.0001		0.0841		0.0434		0.0727
28-29.9 kg/m^2^	74	9.5		24.3		8.1		10.8	
30 - 34.9 kg/m^2^	87	19.5		26.4		12.6		13.8	
≥ 35 kg/m^2^	56	41.1		41.1		23.2		25.0	
									
**Number of associated co-morbid diagnoses**									
**At most two**			0.0056		0.4444		0.0069		0.0169
28-29.9 kg/m^2^	69	10.1		17.4		10.1		13.0	
30 - 34.9 kg/m^2^	80	16.3		21.3		13.8		20.0	
≥ 35 kg/m^2^	56	32.1		26.8		30.4		33.9	
**Three or more**			<.0001		0.0932		0.0104		0.0006
28-29.9 kg/m^2^	85	14.1		27.1		16.5		12.9	
30 - 34.9 kg/m^2^	106	25.5		29.3		16.0		18.9	
≥ 35 kg/m^2^	98	48.0		40.8		31.6		35.7	
									
**Previous efforts for substantial changes in dietary habits (4)**									
**No**			0.0001		0.2115		0.0313		<.0001
28-29.9 kg/m^2^	100	15.0		24.0		13.0		13.0	
30 - 34.9 kg/m^2^	109	23.9		33.0		15.6		18.4	
≥ 35 kg/m^2^	89	41.6		34.8		27.0		38.2	
**Yes**			<.0001		0.0109		0.0072		0.1299
28-29.9 kg/m^2^	50	8.0		22.0		16.0		14.0	
30 - 34.9 kg/m^2^	70	15.7		14.3		15.7		21.4	
≥ 35 kg/m^2^	60	45.0		36.7		36.7		30.0	
									
**Previous efforts for substantial changes in physical activities**									
**No**			<.0001		0.2019		0.0002		<.0001
28-29.9 kg/m^2^	119	14.3		26.9		12.6		13.5	
30 - 34.9 kg/m^2^	145	22.8		29.0		15.2		20.0	
≥ 35 kg/m^2^	122	44.3		36.9		32.0		36.1	
**Yes**			0.0097		0.2248		0.6963		0.2165
28-29.9 kg/m^2^	28	3.6		10.7		21.4		14.3	
30 - 34.9 kg/m^2^	28	10.7		14.3		21.4		21.4	
≥ 35 kg/m^2^	23	34.8		30.4		30.4		34.8	

(1) "I feel I am being attacked when people talk about my weight" (2) "I feel very ill-at-ease " (3) Q25% = first quartile- A score ≤ Q25% indicates a poor QOL, and a high QOL (4) Significant interaction with vitality scores: p = 0.02

### 4- Multivariate models

A BMI of 35 kg/m^2 ^and over was a major correlate of poor QOL for dimensions 1, 3 and 4. Older patients were more likely to experience poorer physical functioning, compared to those under 60. By contrast, these patients showed better results for dimension 3 (Relations with others). Men had a lower risk of impaired psychological well-being (dimension 4). No significant impact on QOL was observed in multivatiate analyses for current smoking, alcohol drinking or co-morbid diagnoses (Table 5). Compared, with workmen and employees, top executives and professional persons had a significantly better QOL for dimensions 1 and 2.

**Table 5:** Risks of poorer QOL (logistic regression models)

	**Dimension 1-** **Physical state (n = 411)**			**Dimension 2 -** **Vitality, desire to do things (n = 395)**		**Dimension 3 -** **Relations with others (n = 412)**		**Dimension 4 -** **Psychological state (n = 428)**	
	**OR**	**95%CI**		**OR**	**95%CI**	**OR**	**95%CI**	**OR**	**95%CI**
**Age (years)**									-
< 60	1.00	-		1.00	-	1.00	-	1.00	
60 - 69.9	1.80	0.97-3.35		1.04	0.58-1.86	0.72	0.38-1.37	0.67	0.36-1.27
≥ 70	2.02	1.10-3.70		1.15	0.65-2.03	0.32	0.15-0.69	0.74	0.40-1.36
									
**Male vs. female**	0.90	0.53-1.51		0.86	0.53-1.40	0.59	0.31-1.12	0.46	0.25-0.82
									
**Body Mass Index (Kg/m^2^)**									
< 29.9	1.00	-		1.00	-	1.00	-	1.00	-
30 - 34.9	1.95	0.98-3.88		1.34	0.75-2.38	1.53	0.72-3.28	1.75	0.89-3.46
≥ 35	5.37	2.72-10.6		1.66	0.92-3.01	3.67	1.77-7.61	3.00	1.54-5.85
									
**Socio-economic status**									
Workmen/employees	1.00	-		1.00	-	1.00	-	1.00	-
Top executives -Professional persons	0.35	0.15-0.81		0.47	0.23-0.95	1.14	0.52-2.46	0.49	0.21-1.14
Teachers/Artisans/shopkeeper	1.00	0.48-2.08		1.06	0.54-2.10	0.91	0.36-2.29	0.93	0.43-2.01
Unemployed/housewives-husbands	1.43	0.75-2.74		0.89	0.47-1.68	1.69	0.84-3.40	1.32	0.69-2.53
									
**Number of associated co-morbid diagnoses**									
None or one	1.00	-		1.00	-	-	-	-	-
Two	1.32	0.56-3.12	-	0.85	0.40-1.81	-	-	-	-
Three or more	1.69	0.79-3.61	-	1.42	0.74-2.75	-	-	-	-
									
**Previous efforts for substantial changes in physical activities**	0.44	0.20-0.94	-	0.50	0.24-1.01	-	-	-	-
									
**Current Smoking (yes/no)**	-	-		1.76	0.91-3.37	1.31	0.63-2.74	-	-
									
**Alcohol**	-	-		-	-				
None	-	-		-	-	1.00	-	1.00	-
1-2 glasses per day	-	-		-	-	1.05	0.53-2.08	0.93	0.50-1.73
≥ 3 glasses per day	-	-		-	-	0.89	0.39-2.11	0.53	0.21-1.33

## Discussion

This is one of the few surveys conducted in pharmacies and investigating the quality of life (QOL) of pre-obese and obese patients. Our data suggest that a BMI of 35 kg/m^2 ^or over had a significant impact on three domains of the OSQOL questionnaire. A minority of patients reported previous efforts to change substantially their habits regarding diet and/or physical exercise. It was observed that older overweight patients exhibited poorer physical functioning than younger patients, whereas their psychological well-being was better. Women's psychological status was more affected than men's, and overall, a better QOL was observed in persons coming from a higher social class.

The lower physical functioning observed with elevated BMI confirms conclusions of previous surveys [12-15]. This result may be partly explained by the osteo-articular and respiratory consequences of excess weight [15]. Relational or psychological consequences should not be overlooked either [17] as common mental distress among obese patients has been observed [18]. Here again, the QOL linked to relational and psychological dimensions significantly decreased with increasing BMI, although less markedly (Table 5). The limited efforts reported by patients to change dietary habits and more specifically to increase exercising are consistent with other authors' conclusions [19].

Patients' QOL significantly decreased with severe obesity (BMI ≥ 35 kg/m^2^) for 3 dimensions of the OSQOL questionnaire (Table 5). Moreover, physical status (dimension 1) was more specifically affected than other dimensions. This finding is consistent with other authors' conclusions [12-14]. However, a significant effect was also retrieved for the mental dimensions (Table 5). Additionally, our findings suggest that the relationships identified between BMI and the different OSQOL dimensions did not substantially vary according to the other factors (Table 4). The only significant interaction identified could not be easily interpreted in concrete terms. Nonetheless, these results require confirmation in further studies.

Patients aged 70 and over presented an increased risk of a worse physical state compared to younger pre-obese and obese patients in multivariate analyses (Table 5). This result may be due to the natural consequences of ageing on physical agility and mobility, irrespective of overweight severity. Our data suggest that older patients are less affected in their relationships by their excess weight. Physical appearance might play a more important role in the social life among younger patients. Indeed, excess weight may be a barrier to developing social activities in younger patients. In contrast, older overweight patients may have become accustomed to their appearance meaning that the impact on their social life is much less important. However, these hypotheses require confirmation and, more generally a better understanding of the effects of age on relationship domain and other QOL dimensions is desirable.

Overall, pre-obese and obese women had a lower QOL than men, notably for relational and psychological dimensions, which is consistent with conclusions of previous studies [13,14,20] with differences being significant only for psychological state in our data (Table 5). The importance of physical appearance for women may explain these results and could account for their lower psychological well-being. In a previous study, obese women ranked their dissatisfaction with physical appearance higher than men [17].

The QOL of professional persons and top executives tended to be better compared to the rest of the study population, although no significant difference was found in the multivariate analysis for relational and psychological dimensions (Table 5). The beneficial impact on QOL of a high social level has been previously described [20] QOL could be partly influenced by the ability to maintain a healthier lifestyle, whether these patients already had a healthy lifestyle or changed their habits due to their excess weight. The links between a lower socioeconomic level, unhealthy eating [21,22] and physical inactivity [23] have also been established.

The beneficial consequences of physical activity on QOL in the context of obesity are well established [14,24]. Regular exercising provides physical and psychological well-being to patients regardless of the severity of their excess weight [24]. This was confirmed by our results where patients who reported efforts to increase physical activity presented significantly improved physical functioning. By contrast, patients with an advanced deteriorated physical state are generally less likely to take on physical activities. Although the influence of co-morbid diagnoses on QOL has been reported in patients with severe obesity [25], no significant effect on QOL could be observed in our study in multivariate analyses.

The impact of smoking habits on QOL was not confirmed (Table 5). The consequences of smoking on physical state are however well established. Our findings should not be over-interpreted, especially as detailed smoking history and smoking years were not documented. Additionally, some patients with significantly impaired QOL may have quit smoking as there were few smokers included in our survey. Likewise, interpretation in our data of the influence on QOL of alcohol requires most caution as the reliability of reported alcohol drinking habits may be questionable, even in an anonymous self-completed-questionnaire.

This study had some limitations. Firstly, we used a convenience sample, which may not be representative of the overall population of pre-obese and obese subjects. Only patients presenting a probable excess weight according to the pharmacist's judgement were asked to participate, meaning that some patients who may have actually met the inclusion criteria were not offered the study. Additionally, our study population recruited in community-pharmacies may present more associated diseases than a more representative sample of pre-obese and obese patients may. Nonetheless, we believe that such a selection bias may not substantially affect our findings: no significant influence of the number of co-morbid diagnoses was noted for physical and vitality scores in multivariate analysis (Table 5) and for the other dimensions in univariate analyses (Table 3).

All data collected on questionnaires about the patients were purely self-reported and the data obtained for reported physical activity and dietary habits should therefore be interpreted cautiously. Further investigations with more accurate assessments of patients' lifestyle should be needed for more conclusive results. In addition, co-morbid diagnoses were identified from drugs dispensed before inclusion and not using specific clinical criteria. As a consequence, diseases not treated by the studied drugs classes (Methods) were not identified and psychiatric co-morbid diagnoses were not evaluated either. Socioeconomic level was assessed on occupational status and it may therefore have been of interest to have complementary data on education or income level. The survey only evaluated a small number of the consequences of excess weight on QOL, and several outcomes of interest in obesity such as sexual life [17,26], detailed eating habits [17], medical supervision, perception of weight status and history of weight loss [27] were not explored. Domains referring to relations with others, psychological distress were only partially studied as only a single item was dedicated to these dimensions in the OSQOL. Given the prominent role of psychological welfare in QOL [13], further studies, with more elaborated instruments are needed to investigate these topics more accurately. Lastly, refusals were not documented. However, as a prerequisite to participate was to be a regular customer of the pharmacy, refusal rate may be assumed to be low.

An originality of the PRICARDO pharmacy-based study was its design. Studies on chronic diseases have been successfully conducted in pharmacies. Pharmacists with whom patients have often built a relationship of confidence are ideally positioned to conduct such studies, notably in case of regular or chronic therapy. Our results proved the feasibility of such a study in the context of pre-obesity and obesity although patients needing a regular treatment are more likely to be easily captured in pharmacies.

The results of this study do have practical implications. Firstly, our findings highlight the clear influence BMI of 35 kg/m^2 ^or over, age, gender and socioeconomic status on QOL of pre-obese and obese patients. These factors should be better considered before investigating and interpreting the QOL in this population. In addition to patients' physical health, consequences on psychological well-being and social life should not be overlooked. Descriptive findings have suggested that the lifestyle of these patients could be improved: educational actions should be implemented to encourage overweight adults to take up physical activities. It will also be important to further understand the dietary habits and patients' reluctance to change their lifestyle. Several interventional studies have actually highlighted the beneficial impact of educational training based on physical activity and an improved compliance to diets in obese patients [28].

## Conclusion

In conclusion, this survey has proved that the consequences of excess weight on patients' lives can be evaluated by studies performed in community-based pharmacies, as already experimented for chronic diseases. Factors such as age, gender, dietary habits, physical activity or socioeconomic level should be more taken into account by care-givers before interpreting QOL in overweight and obese patients.

## Competing interests

The authors declare that they have no competing interests.

## Authors' contributions

GC conceived of the study, its design including questionnaires. She was responsible for pharmacists' recruitment in the study and its global coordination, notably the collection of data. She also participated in the draft. EVG actively participated in the design of the study and in the draft of the manuscript. LLa directed the statistical analyses and drafted the manuscript in the close collaboration of other authors.

CR performed the statistical analyses and participated in the drafting of the manuscript. LLe GD and AM actively participated to the draft with helpful suggestions, both in interpretation of the data, and in suggestions of references

SR read the draft and made significant corrections of English. HM participated to the draft. All authors have read and approved the manuscript

## Pre-publication history

The pre-publication history for this paper can be accessed here:


